# Manipulating T-cell metabolism to enhance immunotherapy in solid tumor

**DOI:** 10.3389/fimmu.2022.1090429

**Published:** 2022-12-22

**Authors:** Chen Chen, Zehua Wang, Yi Ding, Yanru Qin

**Affiliations:** Department of Oncology, The First Affiliated Hospital of Zhengzhou University, Zhengzhou, China

**Keywords:** T cell, metabolism reprogramming, solid tumor, immune checkpoint inhibitor, adoptive cell therapy, oncolytic virus therapy

## Abstract

Cellular metabolism is not only essential for tumor cells to sustain their rapid growth and proliferation, but also crucial to maintain T cell fitness and robust immunity. Dysregulated metabolism has been recognized as a hallmark of cancer, which provides survival advantages for tumor cells under stress conditions. Also, emerging evidence suggests that metabolic reprogramming impacts the activation, differentiation, function, and exhaustion of T cells. Normal stimulation of resting T cells promotes the conversion of catabolic and oxidative metabolism to aerobic glycolysis in effector T cells, and subsequently back to oxidative metabolism in memory T cells. These metabolic transitions profoundly affect the trajectories of T-cell differentiation and fate. However, these metabolic events of T cells could be dysregulated by their interplays with tumor or the tumor microenvironment (TME). Importantly, metabolic competition in the tumor ecosystem is a new mechanism resulting in strong suppression of effector T cells. It is appreciated that targeting metabolic reprogramming is a promising way to disrupt the hypermetabolic state of tumor cells and enhance the capacity of immune cells to obtain nutrients. Furthermore, immunotherapies, such as immune checkpoint inhibitor (ICI), adoptive cell therapy (ACT), and oncolytic virus (OV) therapy, have significantly refashioned the clinical management of solid tumors, they are not sufficiently effective for all patients. Understanding how immunotherapy affects T cell metabolism provides a bright avenue to better modulate T cell anti-tumor response. In this review, we provide an overview of the cellular metabolism of tumor and T cells, provide evidence on their dynamic interaction, highlight how metabolic reprogramming of tumor and T cells regulate the anti-tumor responses, describe T cell metabolic patterns in the context of ICI, ACT, and OV, and propose hypothetical combination strategies to favor potent T cell functionality.

## 1 Introduction

Cellular metabolism drives a variety of biochemical pathways that convert nutrients to small molecular metabolites, comprising three fundamental building blocks: glucose, fatty acid, and amino acid (1). It is well known metabolism is crucial to regulate the phenotype and biological function of cells (2). Given the infinite proliferation of solid tumors that require faster energy supply, they need to reprogram their metabolic patterns in adaption to external stresses, leading to a hypoxia, acidic, and nutrient-deficient tumor microenvironment (TME). A hallmark of cancer is dysregulated metabolism (3–5). As first observed by Otto Warburg, tumor cells display enhanced aerobic glycolysis but decreased oxidative phosphorylation (OXPHOS) even in the presence of oxygen, known as “Warburg effect” or “aerobic glycolysis” (6–8). Apart from glucose metabolism rewiring, lipid metabolism (9, 10), amino acid metabolism (11, 12), and other metabolic pathways (13, 14) are frequently altered in tumorigenesis and cancer progression. These metabolic alterations provide selective advantages for tumor survival, proliferation, invasion, and metastasis (15).

T cells are able to sense detrimental signals and motivate specific immune responses against tumor cells. Emerging evidence revealed that different T cells display different metabolic characteristics. In the normal physiology, metabolic transition profoundly influences the trajectory of differentiation and fate of T cells. For example, the stimulation of resting T cells promotes the conversion of catabolic and oxidative metabolism to aerobic glycolysis in effector T cells, and subsequently back to oxidative metabolism in memory T cells (16–18). However, these transitions could be disrupted by their interplays with tumor or the TME. First, cancer metabolism contributes to a highly acidic, hypoxic, nutrient-deficient, and oxidatively stressed TME, further aggravating metabolic barriers in immunocytes (19–21). Second, competition for nutrients between tumor cell and T cells is a new immunosuppressive mechanism. Tumor cells are capable of competing with immune cells for essential nutrients, thereby reducing the metabolic fitness of tumor-infiltrating immune cells (TILs) and hindering anti-tumor immune response (22, 23). Therefore, metabolic disturbance in the TME might inform how to retrieve anti-tumor response of effector T cells.

Anti-tumor immunotherapies based on immune checkpoint inhibitor (ICI), adoptive cell therapy (ACT), and oncolytic viruses (OV), have greatly revolutionized the clinical management of solid tumor, empowering T cell response to eliminate cancer cells and inducing durable remissions (24, 25). But not all patients respond to these novel agents. The complex integration of T cell metabolism within the TME may contribute to the failure of these immune approaches. In this case, understanding and modulating T cell metabolism may overcome metabolic barriers and provide a bright avenue to improve immunotherapy.

In this review, we provide an overview of cellular metabolism of tumor and T cells, highlight how metabolic reprogramming of tumor and T cells regulate the anti-tumor responses, describe T cell metabolic pattern in the context of ICI, ACT, and OV, and propose hypothetical combination strategies between metabolic intervention and immunotherapy.

## 2 Overview of metabolism in tumor cells and immune cells

### 2.1 Metabolism of tumor cells

Metabolic reprogramming is considered as a hallmark of cancer (6). Tumor cells undergo metabolic adaptions to fuel their infinite division and exhibit altered metabolic pattern compared to normal cells. The initial finding is that tumor cells preferentially use glycolysis to generate ATP along with increased lactate secretion even with abundant oxygen supply, known as ‘Warburg effect’ or ‘aerobic glycolysis’ (26, 27). Meanwhile, tumor cells also uptake a large amount of nutrients, such as glutamine, arginine, tryptophan, and fatty acids to generate ATP and biosynthetic precursors for anabolism (28). Metabolism reprogramming renders tumor cells to better counteract external stresses, most notably the hypoxia and nutrient starvation. Accordingly, these altered metabolic activities and bioenergetic mechanisms are critical to the tumor survival (29).

Tumor cells are in tight interaction with the TME and metabolism reprogramming is affected by both external and internal stimuli (30). Specifically, metabolism provides survival advantages for tumor cells according to the following aspects. First, enhanced glycolysis in tumor cells contributes to the generation of glycolytic intermediates, which in turn favor the pentose phosphate pathway (PPP) pathway, amino acid biosynthesis, and nucleotide biosynthesis (29). Second, tumor cells generate acetyl-CoA by digesting small-chain fatty acids. Acetyl-CoA is conducive to the production of tricarboxylic acid (TCA) cycle intermediates, which are usually used as precursors for macromolecule synthesis, such as fatty acid and cholesterol (31). Third, under the limited nutrient and oxygen supply, tumor cells evolved several mechanisms to sustain the ATP/ADP ratio. Additionally, despite the heterogeneous nature of solid tumor, malignancy seems to activate a series of common oncogenic pathways to support anabolism and redox balance, such as PI3K pathway (30). Taken together, tumor cells always choose the optimal metabolic pattern to satisfy their energic and anabolic demands of enhanced cell proliferation, which partially determine the nutrient and oxygen availability in the TME.

### 2.2 Metabolism of T cells

T cells are at rest when the body is in immune equilibrium, while they could be quickly activated and respond to detrimental infection, inflammation, and tumor-associated antigens (32). There are two main categories of metabolic patterns of T cells: 1) the activated T cells prefer to perform aerobic glycolysis that similarly occurs in cancer cells; 2) the resting T cells mainly depends on TCA and OXPHOS to obtain energy.

**Figure 1:**
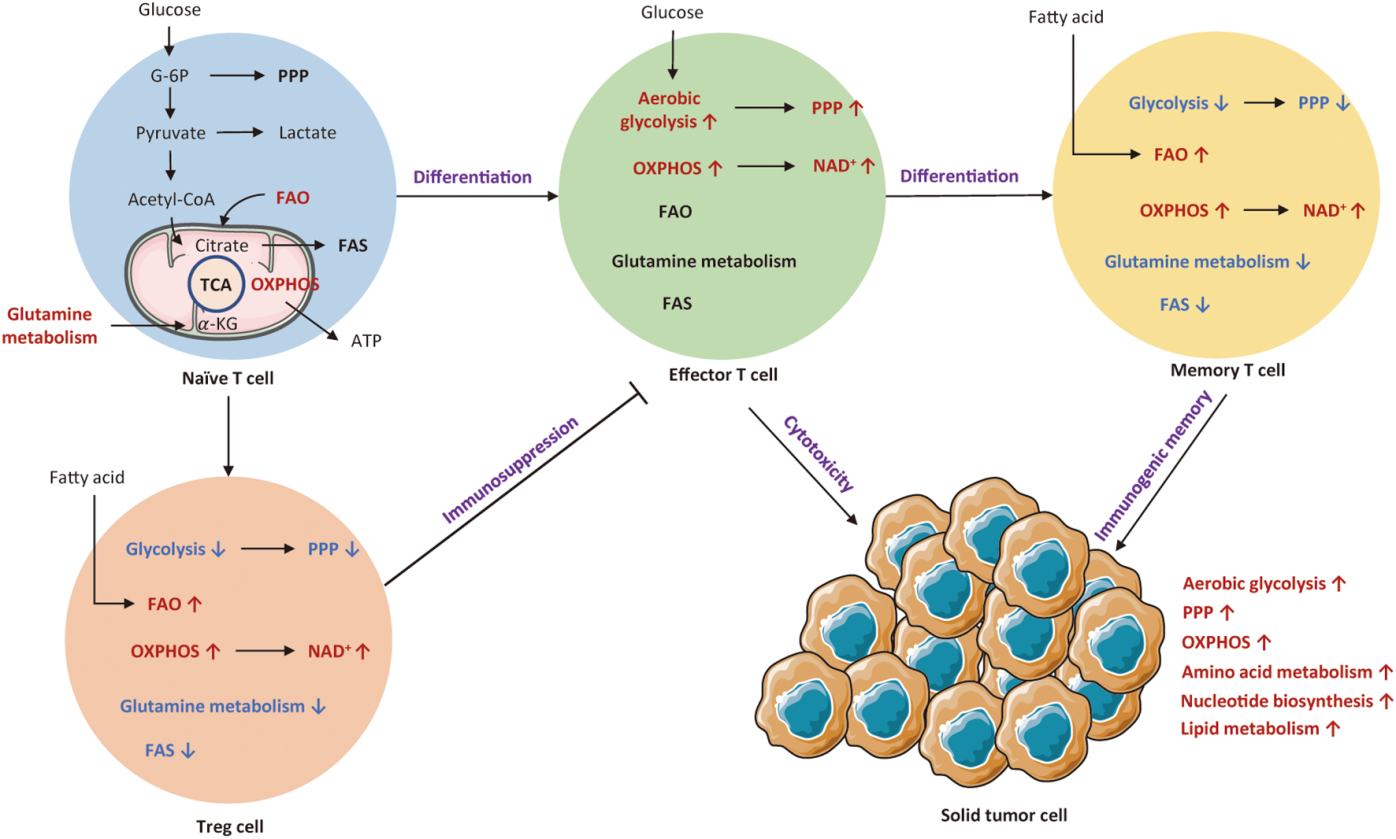
An overview of cellular metabolic pathways of different T subsets. Naïve T cell mainly rely on OXPHOS to maintain minimum ATP levels. Upon activation of T cell receptor (TCR) and co-stimulatory signals, naïve T cells shift to effector T cells that are reliant on aerobic glycolysis and OXPHOS. Both Treg cells and memory T cells mainly rely on OXPHOS and FAO for survival. The red arrows and texts represent enhanced metabolism processes, while the blue arrows and texts represent inhibited metabolic pathways. PPP, pentose phosphate pathway; OXPHOS, oxidative phosphorylation; FAO, fatty acid oxidation; FAS, fatty acid synthesis; TCA, tricarboxylic acid; G-6P; glucose-6-phosphate; Treg cell, regulatory T cells.

T cells are the most important immune cells, which could attack tumor cells or indirectly secrete biological molecules. A recent research indicates that T cell metabolism is closely associated with T cell differentiation, activation, function, memory, and exhaustion (33, 34). Theoretically, resting T cells mainly depend on OXPHOS, while activated T cells display enhanced aerobic glycolysis, glutamine deposition, and reduced fatty acid oxidation (FAO) to meet the requirement of energy. **Figure 1** depicts metabolic transitions during the differentiation from naïve T cell to effector T cell, memory T cell, and regulatory T cell.

It has been known that T cells undergo metabolic reprogramming during activation, which is associated with distinct T cell subsets and functional fates (35). Naïve T cells intake basic nutrient to preserve minimum cellular ATP levels *via* OXPHOS (36). These quiescent T cells also rely on FAO and glutamine metabolism to maintain basic metabolic needs (37, 38). Unlike tumor cells, T cell metabolic reprogramming is initiated by T cell receptor (TCR) activation with co-stimulation (39). Upon encounter with antigen, naïve T cell rapidly shifts to effector T cell that appears as a metabolic activation state with increased nutrient absorption and glycolysis rate (36). It has been shown that effector T cells mainly depend on aerobic glycolysis and OXPHOS to maintain T cell fitness and function (40, 41). Such a metabolic transition involves alterations in cellular signaling and metabolic pathways for optimization of immune functionality and proliferation (42). First, such a metabolic transition significantly upregulates cellular signaling pathways, such as PI3K/AKT/mTOR and Ras/MEK/ERK pathways that lead to the expression of transcription factors like HIF-1*α* and C-MYC, regulating T cell metabolic programs and functional fates (43). Second, these activated T cells experience a similar shift from catabolism toward anabolism, with increased generation of biosynthetic intermediates, including protein, lipid, and nucleotide (44). Eventually, T cells gain the ability to proliferate and generate progeny cells to exert immune functions, secreting inflammatory cytokines and lytic molecules (42). Metabolically, glycolysis produces ATP much faster and offers more nutrients for T cell activation. Therefore, in the differentiation of T cells towards terminal effector status, they become more dependent on glycolysis (45), of which process is similar to the aerobic glycolysis (Warburg effect) long observed in cancer (46).

In parallel to upregulating glycolysis, T cells also engage in rapid uptake and metabolism of glutamine, which enters the tricarboxylic acid (TCA) cycle as *α*-ketoglutarate (47). Glutamine can support OXPHOS *via* the TCA cycle (48). Moreover, both resting and activated T cells consume exogenous lipids and catalyze the oxidation of lipids (44, 49). *β*-oxidation is a highly efficient source to generate ATP. Therefore, the activated T cells can fuel their proliferation by consuming multiple nutrients, forming a superior clonal population with effector functions. Notably, many alterations occurred in rapidly proliferating T cells resemble the process of cancer metabolic reprogramming.

It is well known that the activation of T cells is induced by inflammation and cytokines to acquire different effector functions, thereby metabolic program differs in each subset (50). If these metabolic transitions fail to establish, T cells cannot exert their particular functions and instead differentiate into regulatory T cells (Treg). Differently, Treg cells mainly rely on OXPHOS and FAO to supply ATP. Such a metabolic profile endows the TME with immunosuppressive property (51). After antigen clearance, memory T cells establish immunological memory to allow for rapid activation when encountered with the same antigen (52). The metabolic pattern of memory T cells is similar to that of naïve T cells, with reliance on OXPHOS and FAO for basic energy (53, 54). If antigens are not quickly cleared, persistent antigen exposure would result in T cell dysfunction (55). Unlike memory T cell, exhausted T cells show dampened proliferative capacity and marked metabolic dysfunction. Specifically, glucose uptake and OXPHOS are decreased, whereas mitochondrial mass is increased (56).

In summary, different metabolic patterns influence the differentiation of various T cell subtypes. **Table 1** summarizes metabolic characteristics of distinct T cell populations. Exploring the metabolic reprogramming of T cells could help us to better understand the essence of immune responses.

**Table 1:** An overview of metabolic characteristics of different T cell populations.

Immune cell	Subtypes	Metabolic pattern	Function	Reference
T cell	Naïve T cell	OXPHOS, FAO, Glutamine metabolism	Proliferation and differentiation	(36–38)
	Effector T cell	Aerobic glycolysis, OXPHOS	Cytotoxicity	(36, 40, 41)
	Memory T cell	OXPHOS, FAO	Immunological memory	(53, 54)
	Regulatory T cell	OXPHOS, FAO	Immune suppression	(51)

OXPHOS, oxidative phosphorylation; FAO, fatty acid oxidation.

## 3 Metabolic relationship between the tumor cells and T cells

The metabolic and nutrient changes in TME could reshape metabolic programming of T cells by inhibiting T cell differentiation and enhancing immunosuppression. Hypermetabolic cancer cells could induce nutrient deprivation. This condition would impair TCR signaling, glycolysis, and amino acid metabolism, and consequently impose stress on the tumor-specific T cells infiltrating into the TME (57). Metabolic changes of the TME affect immune cell activation and proliferation. Targeting these metabolic changes can motivate novel immunometabolism strategies. Details have been summarized in **Table 2**.

**Table 2:** Effects of metabolic changes of the TME on immune cell function and potential immunometabolism therapies.

Factor	TME	Immune cell		Immunometabolism therapy			Reference
	Metabolite changes	Altered metabolism	Effect on immunity	Target site	Metabolic modulation	Effect on T cell	
**Nutrient** **depletion**	Glucose ↓	Aerobic glycolysis ↓	CTL function ↓ CTL Apoptosis ↑	Tumor cell	Glycolysis inhibition PD-1 blockade	Increase T cell glycolysis	(58–61)
	Glutamine ↓	Glutamine uptake ↓	CTL function ↓	Tumor cell	Glutamine antagonist Glutamine transporter inhibitor	Increase glutamine availability for CTL	(62–64)
	Tryptophan ↓	Tryptophan uptake ↓	CTL proliferation ↓	TME	IDO inhibition	Increase tryptophan availability for CTL	(65–67)
	Arginine ↓	Arginine uptake ↓	CTL proliferation ↓	TME	Arg1 inhibition	Increase arginine availability for CTL	(68–70)
**PH**	Lactate ↑	Lactate influx ↑	CTL activity ↓	Tumor cell	LDHA inhibition MPC inhibition	Increase cytotoxic activity of CTL	(71–74)
	Acidification	PH ↓	M2 polarization ↑ Treg cell maintenance CTL activity ↓	Tumor cell	MCT inhibition	Increase cytotoxic activity of CTL	(73, 75, 76)
**Oxygen**	Hypoxia	HIF1 activation ↑ OXPHOS ↓	CTL activity ↓ Th17 differentiation ↑	Tumor cell	Metformin ETC inhibition	Supply oxygen to CTL	(77–80)
**ROS**	ROS ↑	ROS accumulation	CTL activity ↓ Treg cell maintenance	T cell	ROS scavenger	Alleviate oxidative stress on CTL	(57, 81–83)
**Metabolites**	Fatty acid ↑	FAO ↑	Treg cell maintenance Memory T cell longevity ↑	TME	AMPK activation	Increase CD8^+^ T cell recruitment and memory differentiation	(84–87)
	Adenosine ↑	Adenosine accumulation	CTL activity ↓ Treg cell maintenance	T cell	A2AR inhibition	Render TME less immunosuppressive; increase cytokine secretion	(88–91)
	Kynurenines ↑	Kynurenine accumulation	CTL proliferation ↓ PD-1 expression ↑ Cytokine secretion ↓ Treg cell maintenance	TME	IDO inhibition Kynureninase	Render TME less immunosuppressive; prompt CTL activity	(65, 92, 93)
	Prostaglandins ↑	Prostaglandins accumulation	CTL activity ↓ Treg cell maintenance	Tumor cell	COX inhibition	Render TME less immunosuppressive; increase cytokine secretion	(94, 95)

TME, tumor microenvironment; OXPHOS, oxidative phosphorylation; FAO, fatty acid oxidation; COX, cyclo-oxygenase; IDO, indoleamine 2,3-dioxygenase; ROS, reactive oxygen species; LDHA, lactate dehydrogenase; A2AR, A2A receptor; CTL, cytotoxic CD8^+^ lymphocytes; Treg, regulatory T cell; M2, macrophage 2; Arg1, arginase 1; MCT, monocarboxylate transporter; ETC, electron transport chain; AMPK, AMP-activated protein kinase; MPC, mitochondrial pyruvate carrier.

### 3.1 Metabolic reprogramming of tumor cell affects T cell function

**Figure 2:**
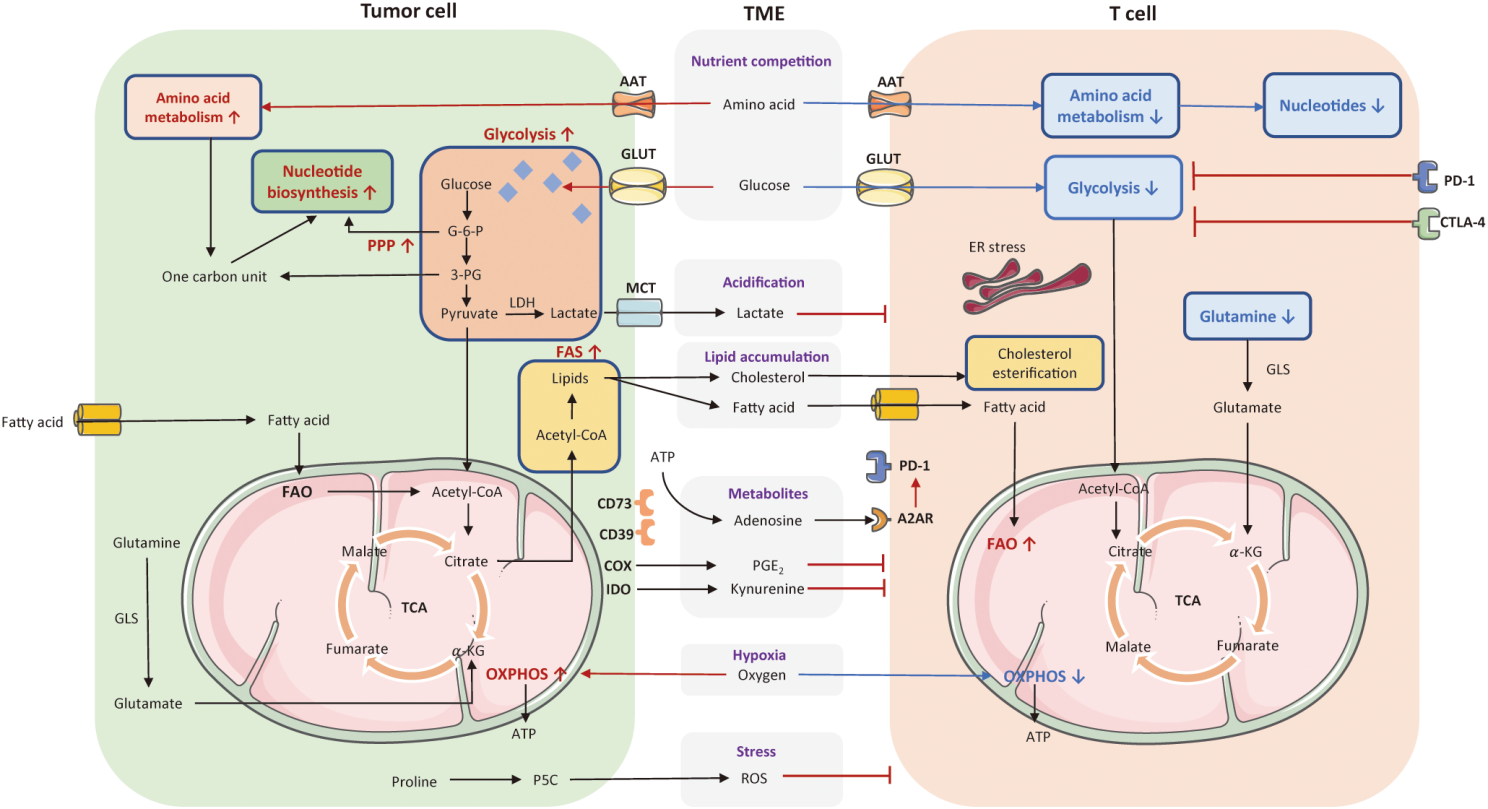
The metabolic communication and competition between tumor cells and T cell. Cellular metabolic disturbances lead to a highly acidic, hypoxic, oxidative stressed, and immunosuppressive tumor microenvironment (TME), which profoundly affect T cell anti-tumor responses. Tumor cells consume a large amount of glucose and amino acids to fuel their rapid proliferation. These pathways greatly limited nutrient and availability to T cells in the TME. The enhanced glycolysis in tumor cells also induce the generation of lactate that can acidify the TME and exert an immunosuppressive effect on T cells. Tumor cells with enhanced FAS lead to lipid accumulation in the TME, including fatty acid and cholesterol, variously affecting T cell function. Also, tumors cells released several cancer metabolites, such as adenosines, kynurenines, prostaglandins, which suppress T cell effector function. Moreover, tumor cells consume a high level of oxygen to favor OXPHOS, contributing to hypoxia in the TME along with the production of ROS, and thereby inhibiting T cell anti-tumor functionality. The red arrows and texts represent enhanced metabolism processes, while the blue arrows and texts represent inhibited metabolic pathways. PPP, pentose phosphate pathway; OXPHOS, oxidative phosphorylation; FAO, fatty acid oxidation; FAS, fatty acid synthesis; TCA, tricarboxylic acid; AAT, amino acid transporter; GLUT, glucose transporter; MCT, monocarboxylate transporter; G-6-P, glucose-6-phosphate; 3-PG, 3-phosphoglyceric acid; LDH, lactate dehydrogenase; GLS, glutaminase; ATP, adenosine triphosphate; A2AR, A2A receptor; PGE_2_, prostaglandins, COX, cyclo-oxygenase; IDO, indoleamine 2,3-dioxygenase; ROS, reactive oxygen species; ER, endoplasmic reticulum; TME, tumor microenvironment; MPC, mitochondrial pyruvate carrier.

Cellular metabolic disturbances lead to a highly acidic, hypoxic, immunosuppressive TME, which is associated with cancer progression and immune escape (96). The metabolic communication and competition between tumor and T cells have been depicted in **Figure 2**.

#### 3.1.1 Acidification

Excessive lactate produced by tumor cells is transported by monocarboxylate transporters (MCTs), with increased acidity of the TME that further interferes with T cell functionality and longevity (97). First, lactate can suppress PI3K/AKT/mTOR pathway, and thereby inhibit T cell glycolysis (71, 72). Second, by downregulating NFAT, lactate reduces IFN-*γ* secretion and curtails anti-tumor immune response. Third, lactate metabolites limit the motility of CD4^+^ and CD8^+^ T cells to infiltrate into solid tumors (73). Finally, acidification of the TME can impair the cytokine production of T cells (74). Other than dysfunctional T effector cell induced by lactate, it has been shown to polarize macrophage into M2 phenotype and prompt metabolic transition of Treg cells to preserve their activity in glucose-deficiency TME (73, 75, 76). An interesting finding is that glycolysis and the TCA cycle are not coupled at the basis of lactate. In mice models with pancreatic and lung cancer, lactate makes a large contribution than glucose to fuel the TCA cycle (98). Of note, TCA metabolites are substantially involved in the regulation of immune response. As a crucial carbon source to the TCA cycle, manipulating lactate metabolism is a potential strategy to counteract hypermetabolic tumors. For example, LDHA inhibitors can neutralize the acidity of TME, prompts CD8^+^ TIL infiltration, reduces carbon sources for TCA cycle, and decreases the number of immunosuppressive cells (99). Pharmacological inhibition of lactate transporters of MCT1 and MCT4 minimizes the accumulation of lactate in the TME as well.

#### 3.1.2 Hypoxia

As the Warburg effect consumes ample oxygen, the TME often keeps anoxia. Under hypoxia conditions, HIF-1α is upregulated in T cells, which subsequently inhibits PGC1*α* activation to damage T-cell anti-tumor function (100–102). High-expression of HIF-1α impairs CD8^+^ TILs, as evidenced by the delay of tumor progression in mice treated with HIF-1α-knockdown CD8^+^ T cells (77). Increased HIF-1 α is able to prompt Th17 differentiation with overexpression of signature genes, such as ROR*γ*T (78). The regulatory or inflammatory characteristic of Th17 differs in the nature of activation stimulus. However, there are contradictory views whether HIF-1α restrains Treg cell differentiation or prompt their immunosuppressive function *in vivo* (79, 80).

#### 3.1.3 ROS

In tumor cells, mitochondrial respiration produces increased reactive oxygen species (ROS) and then releases them into the TME, exerting a detrimental effect on T cell function (57). The rising levels of ROS in T cells impede metabolic transition in T cells by blocking signaling pathways of mTOR, NFAT, and MYC (81). However, Treg cells containing a higher concentration of GSH may develop resistance to ROS accumulation (82). Therefore, rising ROS levels in the TME impair effector T cell function without compromising the development of Treg cells. Importantly, ROS scavengers can restore these defected effector T cells. Research suggests that naïve CD8^+^ T cells cultured with ROS scavengers can differentiate into more memory stem T cells with better tumor control (83).

#### 3.1.4 Tumor metabolites

Both tumor cells and myeloid-derived suppressor cells (MDSC) express CD39 and CD73 which facilitate the hydrolysis of ATP to adenosine (88). Adenosine, an immunosuppressive metabolite accumulated in the TME, poses a threat on T cell function (88). Adenosine receptor, named A2A receptor (A2AR) is highly expressed in T cells among tumor patients (103). The adenosine signaling *via* biding to A2AR can inhibit IL-2R signaling and NF-*ĸ* B pathway in T cells (89). Adenosine also engages in Treg cell proliferation and correspondingly prompts their immunosuppressive activity (90, 91). A2AR inhibitor, such as CPI-144, may address the immunosuppressive effect induced by adenosine (NCT02655822).

Prostaglandin E_2_ (PGE_2_) is a bioactive lipid metabolite catalyzed by cyclo-oxygenase (COX) in tumor cells (104). PGE_2_ accumulating in the TME has immunosuppressive effects. On the one hand, PGE_2_ can suppress IL-2 and IFN-*γ* secretion by CD4^+^ T cells (94). On the other hand, PGE_2_-mediated signaling could increase the expression of Treg-specific marker Foxp3 prompting tumor progression (95). COX 1/2 inhibitor, such as aspirin, may rescue the negative influence of PGE_2_.

The metabolite kynurenine is largely synthesized by indoleamine-2, 3-dioxygenase (IDO) in tryptophan metabolism (105). The expression of SLC7A5 on T cell surface prompts kynurenine influx that constrains T cell functionality (92). Specifically, kynurenine is able to induce PD-1 expression in T cells by activating aryl hydrocarbon receptor (AHR) to inhibit effector T cell proliferation (65). Conversely, T cells can exacerbate kynurenine production in tumor cells (93). IFN-*γ* secreted by T cells can motivate self-renewal tumor cells to release more kynurenines that are further transported into T cells by SLC7A8/PAT4, leading to the overexpression of PD-1 in T cells (93). IDO inhibitor and kynureninase might alleviate the immunosuppressive influence of kynurenine.

### 3.2 Metabolic reprogramming of T cell regulates anti-tumor responses

T cells play pivotal roles in eliminating tumor cells and serve as major mediators in immunotherapy (106–109). Aberrant cellular metabolism is a major cause of immune cell dysfunction (110–112). As metabolic pathways utilized in each subset are distinct, the differential access to nutrients can affect the path of T cell activation, antigen recognition, and cytotoxicity. Recent studies found that disrupted T cell metabolism is frequently along with depleted anti-tumor immunity and impaired homeostasis (113). Therefore, we mainly depict the metabolic pattern of T cells during anti-tumor responses. Metabolism pathways of T cells have been presented in **Figure 2**.

#### 3.2.1 Glucose metabolism

Glycolysis is a basic pathway for T cell activation and function. During the trajectory of T cell differentiation, glucose uptake is gradually enhanced by signaling pathways to maintain normal T cell response (114). However, the lack of glucose would interfere with T cell function.

On the one hand, to satisfy the need for rapid growth, tumor cells consume large amounts of glucose for glycolysis, leading to a glucose-deficient TME that restricts aerobic glycolysis in tumor-infiltrating lymphocytes (TILs). Glucose restriction leads to TIL linked to reduced mTOR activity, following decreased glycolytic capacity and IFN*γ* production. Previous study indicated that CD8^+^ T cells in the glucose-deprived setting, express relatively low levels of effector molecules of perforin and granzymes (58). The TME with low-glucose may also lead to T cell anergy or even apoptosis *via* the Noxa/Mcl-1 axis (59). On the other hand, increased glycolysis in tumor cells would cause vigorous production of lactate, which acidifies the TME and consequently suppresses the proliferation and effector functions of T cells (115). Further, Treg cells are mainly reliant on FAO rather than glycolysis, which could survive under this condition and play immunosuppressive roles (50). Thereby, glucose restriction not only inhibits T cell-mediated anti-tumor response but also boosts Treg cells-mediated immunosuppression, creating opportunity for tumor progression (3, 101). Overall, glucose dearth is one of the major drivers for T cell dysfunction in the TME. Consequently, the enhanced glucose influx of T cells and increased glycolysis in TME might support T cell anti-tumor functionality (60, 61).

CD8^+^ effector T cells utilize glucose-derived pyruvate to fuel both lactate fermentation and mitochondrial oxidation (18). Manipulating pyruvate metabolism holds the potential to orchestrate CD8^+^ T cell differentiation. It is widely recognized that pyruvate is transported into mitochondria by mitochondrial pyruvate carrier (MPC), a heterodimer in the inner mitochondrial membrane (116). Previous study suggested that mitochondrial pyruvate import by the MPC affects thymic development of T cell precursor (117). Importantly, a recent study indicated that genetic deletion of MPC does not influence effector function but favors CD8^+^ T cell differentiation into a memory phenotype (118). Transplantation of CAR-T cells with MPC inhibitors might result in superior and persistent anti-tumor response in preclinical models (118).

#### 3.2.2 Amino acid metabolism

Likewise, amino acids (AA) are heavily utilized by tumor cells, limiting AA availability to T cells in the TME. These classical amino acid metabolic pathways are unbalanced between tumor cells and T cells, which would eventually impair T cell cytotoxic functions.

Glutamine is an essential intermediate to fuel the TCA cycle and generate nucleotides and proteins. Glutamine restriction in the TME hinders effector T cell activation and cytokine secretion but prompts CD8^+^ memory T cell differentiation (62). Besides, tumor cells consumed glutamine to synthesize *γ* -aminobutyric acid that inhibits chemokine expression, such as CCL4 and CCL5, thereby blocking CD8^+^ cytotoxic T cells infiltrating into solid tumors (119, 120). Glutamine antagonists can replenish glutamine availability for T cells and alleviate hypoxia in the TME (63). Additionally, authors showed that glutamine transporter inhibitor could selectively inhibit glutamine uptake by tumor cells, and conversely improve the influx and utilization of glutamine by T cells (64). Overall, tumor cells generally display less plasticity when inhibiting glutamine metabolism, but T cells could be metabolically reprogrammed with stronger survival, proliferation, and cytotoxicity. Blocking glutamine metabolism is a potential strategy to modulate tumor and T cell metabolism, thus overcoming immune escape of tumors (63).

Arginine depletion in the TME weakens the proliferation of activated T cells (68). It has been shown arginine supplementation not only enables the metabolic switch from glycolysis to OXPHOS to maintain the phenotype of central memory T cells, but also enhances the activity of CD8^+^ TILs (121). Both tumor cell and Treg cell utilize arginase enzyme ARG1 to degrade arginine. Therefore, ARG1 inhibitors can revert the availability of arginine in the TME to support effector T cell function (69, 70, 122). One study also demonstrated that the knockout of arginase 2 (ARG2), another enzyme for arginine degradation, can enhance anti-tumor response by coping with unbalanced nutrients (123). Further, tumor cells and T cells may present different states according to ASS1 expression that can facilitate the synthesis of arginine from citrulline. Notably, due to the failure to bind with ATF4 and CEBP*β*, ASS1 is poorly expressed in T cells along with damaged T cell function (124). Evidently, these is a tight association between arginine metabolism and T cell function.

Tryptophan is another AA consumed by tumor cells, leading to inadequate availability of tryptophan for T cells. Tryptophan metabolism is an immunosuppressive factor that prompts tumor progression and accelerates T cell dysfunction, correlating with poor clinical prognosis. As evidenced by a study, ovarian cancer patients with highly active tryptophan metabolism tend to have poor overall survival and disease-free survival (65). Tryptophan degradation is mainly led by indoleamine-2, 3-dioxygenase (IDO1) and tryptophan-2, 3-dioxygenase (TDO2), converting tryptophan into kynurenic acid (125). High levels of IDO and TDO in tumor are thought to reduce tryptophan availability in the TME, limiting the tumoricidal functions of effector T cell (66) and prompting Treg cell generation (67). Overall, IDO inhibition may effectively improve T cell effector function.

#### 3.2.3 Lipid metabolism

Naïve T cells, Treg cells, and memory T cells all need FAO-sourced OXPHOS for survival. Therefore, fatty acid metabolism is important to maintain immunity (126). However, tumor cells occur abnormal lipid metabolism to affect T cell function, which in turn prompts tumor progression (126, 127). Also, excessive lipids in the TME play an immunosuppressive role (77, 84). T cells that highly expressed CD36 can take up more poly-unsaturated fatty acids and oxidize low-density lipoproteins (LDL) accumulated in the TME, eventually impairing anti-tumor responses (85, 128).

Hypermetabolic tumor cells display increased fatty acid synthesis, leading to the accumulation of lipids that exert varying effects on T cell subsets. For Treg cells that uptake exogenous fatty acid to establish their suppressive functions, the incremental lipid content contributes to establishing the immunosuppression TME (50, 86). By contrast, Th17 cells mainly adopt lipogenesis and glycolysis to maintain differentiation. Acetyl-coenzyme A carboxylase (ACC1) is a rate-limiting enzyme in fatty acid anabolism. ACC1 inhibition could promote Treg differentiation but compromise Th17 development (129).

Fatty acid metabolism is critical to effector T cell in the TME. Although effector T cells mainly rely on glycolysis to gain energy, enhanced FAO in CD8^+^ T cells could stabilize anti-tumor function with low glucose and oxygen supply (77, 130). However, some studies put forward a contradictory finding that FAO may prevent the anti-tumor immunity of CD8^+^ T cells (87). A subset of CD8^+^ T cells that take up more lipids could upregulate PD-1 expression, which normally suppress their function. This T cell subset has a highly efficient response upon treatment with PD-1 blockade (131). Similar to the chronic viral infection model, CD8^+^ T cell subsets with distinct expression levels of PD-1 partially differ in their state of exhaustion in the non-small cell lung cancer (NSCLC) model (131). PD-1 in exhausted T cell promotes FAO to restrain anti-tumor response by upregulating STAT3 signaling pathway, which could be recovered by STAT3 signaling inhibitors and FAO inhibitors (87). Additional validation study is still needed to guide precision medicine. Therefore, the effect of FAO on effector T cells is linked to a complicated and controversial metabolism. These contradictory findings partly due to different recruitment of CD8^+^ T cell subsets.

Strikingly, cholesterol also participates in immune cell homeostasis. On the one hand, the high level of cholesterol generated in tumor cells can protect them from immune surveillance (132). On the other hand, in-depth studies demonstrated that altered cholesterol affect metabolic reprogramming of T cells. Explicitly, cholesterol esterification can impair T cell immune responses (133). Disruption of cholesterol esterification to maintain cholesterol concentration in the plasma membrane of immune cells, might potentiate T cell effector function (133). Mounting evidence also suggested that elevated cholesterol in the TME strengthens endoplasmic reticulum (ER) stress in T cells to facilitate the expression of inhibitory immune checkpoint on T cells (134). Moreover, high cholesterol may disrupt lipid metabolism network in T cells, which is detrimental to the immune response (135).

## 4 Metabolic barriers to immunotherapy

Anti-tumor immunotherapies include immune checkpoint inhibitors (ICI), adoptive cell therapy (ACT), and oncolytic virus (OV) therapy. Metabolic barriers underwent by the adaptive immunity to each immunotherapy differ by specific location and immune approach. Each immunotherapy affects T cell response at a various stage. PD-1 blockade prompts CD8^+^ T cell differentiation (136); CTLA-4 blockade solves restrictions on T cell priming and activation (25); ACT may bypass limitations on T cell priming and expansion (137); OV therapy mediates new T cell priming and activation by lysing tumor cells to release tumor-associated antigens (138). However, effector T cells undergo metabolic restrictions upon infiltration into the solid tumors. Most issues are not addressed with current immunotherapies.

**Table 3** summarizes intrinsic and external metabolic barriers experienced by T cells in the context of ICI, ACT, and OV therapy. The intrinsic metabolic challenges are in many aspects. The binding of PD-1 results in the inhibition of glycolysis but the promotion of FAO (139). CTLA-4 ligation inhibits glycolysis during T cell activation (35). CAR-T cells in hyperglycemia conditions of *in vitro* expansion are confronted with metabolic stress after transplantation (45, 141). For OV therapy, initial T cell activation lacks enough metabolic intermediates (143). As discussed above, the TME also coexists and interacts with T cells, bringing metabolic disadvantages to effector T cell. Fortunately, based on combinational therapy or engineering strategy, these immunotherapies all hold the promise to overcome metabolic challenges and improve therapeutic responses.

**Table 3:** The mechanism of metabolic barriers of different immunotherapies.

Immunotherapy	Class	Mechanism of action	Intrinsic metabolic challenges	Extrinsic metabolic challenges	Reference
Immune checkpoint inhibitor	Anti-PD1	Prompt progenitor CD8^+^ T cell differentiation	PD-1 ligation inhibits glycolysis but enhances FAO during effector T cell activation	Hypoxia and low α-KG in the TME negatively affect epigenetic remodeling	(35, 70, 136, 139, 140)
	Anti-CTLA4	Enhance cytolytic CD8^+^ T cell priming; inhibit Treg cells	CTLA-4 ligation inhibits glycolysis upregulation during effector T cell activation	High lactic acid levels support Treg cell function	(25, 35)
Adoptive cell therapy	CAR-T cells	Bypass priming; infiltrate and attack tumor cells	T cells in hyperglycemia culture of vitro expansion may induce metabolic stress after transplantation	Competition for glucose within the TME; hypoxia prevents CAR-T cell infiltration	(45, 62, 137, 141, 142)
Oncolytic virus	T-VEC	Prime new T cells by lysing tumor cells to release TAA	T cells lack metabolic intermediates for initial activation	Hypoxia prevents OV replication and infiltration	(138, 143–145)

T-VEC, talimogene laherparepvec; OV, oncolytic virus; TME, tumor microenvironment; CAR-T, chimeric antigen receptor; α-KG, α-ketoglutarate; FAO, fatty acid oxidation; Treg, regulatory T cells; TAA, tumor-associated antigens.

## 5 T-cell metabolism in the context of ICI

### 5.1 Metabolic effects of immune checkpoints

**Figure 3:**
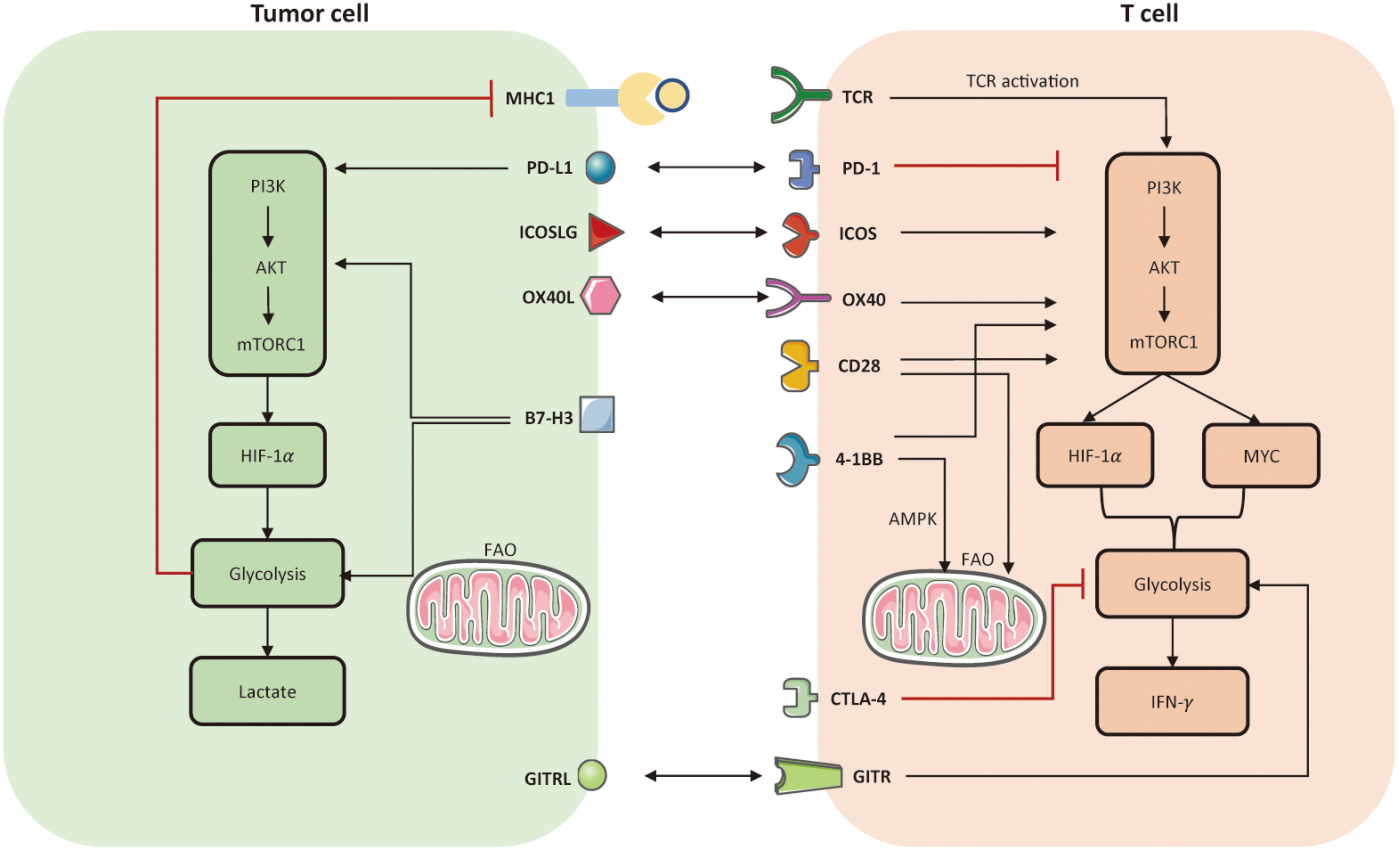
Metabolic modulation of immune checkpoints and their ligands. Activation of TCR and co-stimulatory molecules respectively provides signal 1 and 2 to activate T cell anti-tumor function, referring to the establishment of PI3K-AKT-mTOR, HIF-1*α*, MYC. In tumor cells, PD-L1 and B7-H3 could activate aerobic glycolysis *via* activating PI3K-AKT-mTORC1 pathway. But the enhanced glycolysis limits the expression of MHC-I molecule on tumor cell surface, which may induce immune escape. In T cells, both PD-1 and CTLA-4 signaling impair glycolysis. The difference is that, PD-1 also prompts FAO but CTLA-4 has no effect on FAO. Co-stimulation receptors, such as ICOS, OX-40, CD-28, 4-1BB, all facilitate glycolysis in T cells by activating PI3K-AKT-mTORC1. Notably, 4-1BB is able to enhance mitochondrial activity in CD8^+^ TILs as well. Another co-stimulatory receptor, named glucocorticoid-induced TNFR-related protein (GITR), can prompt nutrient uptake and glycolysis in effector CD8^+^ T cells. FAO, fatty acid oxidation; TCR, T cell receptor.

Indeed, T cells can produce intrinsic metabolic barriers to block their activation, such as inhibitory molecules of PD-1 and CTLA-4. Theoretically, ICI aims to defeat inhibitory signals and further activate endogenous effector T cells, representing an outstanding advance in the treatment of solid tumors. Several studies indicated that ICI may impact the communication and competition between cancer and T cells (139). One study manifested that PD-1 signaling can inhibit glucose uptake and glycolysis by downregulating the PI3K/AKT/mTOR pathway, thereby impairing T cell activation (139). Conversely, PD-1 signaling favors FAO by upregulating AMPK activity and stimulating CPTA1 expression (35). It is notable CTLA-4 signaling also inhibits glycolysis but has no effect on the rate of FAO (35). Apart from metabolic modulation of TILs, immune checkpoints also directly affect cancer metabolic programming. For instance, PD-L1 and B7-H3 could enhance aerobic glycolysis in tumor cells by activating PI3K-AKT-mTOR pathway and HIF-1*α* (146). Therefore, inhibition of PD1-PD-L1 axis may generate a synergistic effect to boost anti-tumor immunity, simultaneously reinvigorating metabolic fitness of TILs and inhibiting aberrant metabolic profiles of tumor cells. Co-stimulatory receptors, such as CD28, 4-1BB, OX-40, ICOS, and GITR also participate in metabolic reprogramming to support T cell activation. For instance, CD28 co-stimulation mainly prompts aerobic glycolysis (147) and facilitates mitochondrial morphology (148–150); 4-1BB co-stimulation mainly activates FAO and mitochondrial biogenesis (60). Metabolic modulation of immune checkpoints and their corresponding ligands are presented in **Figure 3**.

Overall, as both PD-1 and CTLA-4 suppress T cell glycolysis that is necessary for T cell activation, both PD1-targeted ICI and CTLA4-targted ICI could prompt glucose influx and glycolysis to enhance effector T cell function. Ongoing clinical trials combining metabolic interventions and ICI have been summarized in **Table 4**.

**Table 4:** Ongoing clinical trials combining metabolic interventions and immune checkpoint inhibitors.

Metabolic target	Metabolic drug	Immune blockade	Tumor type	Phase	Status	Enrollment	Identifier
Glucose metabolism	Metformin	Pembrolizumab	Melanoma	I	Recruiting	30	NCT03311308
		Nivolumab	Colorectal adenocarcinoma	II	Active	24	NCT03800602
Glutamate pathway	CB-839	Nivolumab	RCC, NSCLC	I-II	Completed	118	NCT02771626
	BHV-4157	Nivolumab/Pembrolizumab	Solid tumor	II	Completed	14	NCT03229278
Arginine pathway	INCB001158	Pembrolizumab	Solid tumor	I-II	Completed	260	NCT02903914
	ADI-PEG 20	Pembrolizumab	Solid tumor	I	Terminated	33	NCT03254732
IDO inhibitors	Epacadostat	Pembrolizumab	UC	III	Completed	93	NCT03361865
	Epacadostat	Pembrolizumab	NSCLC	II	Completed	154	NCT03322540
	Epacadostat	Pembrolizumab	NSCLC	II	Completed	233	NCT03322566
	BMS-986205	Nivolumab	Melanoma	I-II	Completed	627	NCT02658890
	GDC-0919	Atezolizumab	Solid tumor	I	Completed	158	NCT02471846
	HTI-1090	Camrelizumab	Solid tumor	I	Active	23	NCT03491631
COX inhibitors	Aspirin	Pembrolizumab	HNSCC	I	Recruiting	20	NCT03245489
Adenosine pathway	Oleclumab	Durvalumab	Ovarian cancer	II	Completed	25	NCT03267589
	A2AR antagonist: PBF-509	Spartalizumab	NSCLC	I-II	Completed	92	NCT02403193
	A2AR antagonist: CPI-444	Atezolizumab	Solid tumor	I	Completed	502	NCT02655822
	A2AR antagonist: AZD4635	Durvalumab	Solid tumor	I	Active	313	NCT02740985

COX, cyclooxygenase; IDO, indoleamine; NSCLC, non-small-cell lung carcinoma; RCC, renal cell carcinoma; HNSCC, head and neck squamous cell carcinoma; A2AR, adenosine receptor A2A; UC, Urothelial carcinoma.

### 5.2 ICI may overcome metabolic barriers imposed by the TME

As previously discussed, TME imposes a variety of extrinsic metabolic barriers on TILs, such as mitochondrial dysfunction, hypoxia, and acidification (148). These metabolic barriers may be overcome by ICI.

Mitochondria profoundly affect T cell survival and function. It has been found the activation of transcriptional activator PGC1*α* is essential to mitochondrial biogenesis (151). Specifically, the loss of mitochondrial mass might be the consequence of durable AKT activation followed by downregulation of PGC1*α*, which co-activates transcriptional factors, such as PPAR*γ*, NRF1, NRF2, and ERR*α*, and thereby motivates mitochondrial biogenesis and FAO (151). One can assume that overexpression PGC1*α via* 4-1BB stimulation may revert mitochondrial dysfunction and prevent T cell exhaustion (152). Importantly, the combination of anti-PD1 blockade and PPAR- PGC1*α* agonist (bezafibrate) can increase mitochondrial biogenesis, FAO, and OXPHOS in CD8^+^ TILs (130). The hyperpolarized mitochondria also contribute to the generation of ROS, which can promote the exhausted T cell phenotype through epigenetic remodeling (153–155). More recently it has been shown ROS scavengers can synergize with ICI and improve anti-tumor functionality (153–155).

The hypoxic TME limits the oxidative capacity of TILs. There is a high probability that tumor with enhanced oxidative metabolism would fail to respond to PD-1 blockade (140). It is anticipated that supplemental oxygen might boost ICI responses. For instance, the supply of oxygen could reduce the amount of immunosuppressive adenosine in mice model transplanted with lung cancer, along with increased CD8^+^ TILs and decreased Treg cells (69). Additionally, metformin, a drug already used for diabetic patients, is able to inhibit oxygen consumption by tumor cells, alleviating the TME hypoxia. A strong evidence manifested that metformin and ICI have a synergistic effect to improve T-cell function and enhance tumor clearance (70). In a mouse model treated with metformin, tumor hypoxia is alleviated and the response to anti-PD1 blockade is greatly improved (153). Altogether, oxygen deficiency either disturbs normal T cell activation or supports T cell exhaustion, which may contribute to the resistance to ICI. Therefore, identifying the tumor metabolic pattern of each patient may help to inform combinational treatment.

Accumulation of lactate in the TME contributes to a higher tumor burden among patients (115). A prior research found that the TME at neutral PH can potentiate the efficacy of ICI (156). Diclofenac has been identified as an MCT inhibitor (157). Combinational therapy of diclofenac and ICI in mice model showed a promising efficacy profile with prolonged survival (97).

### 5.3 ICI may overcome nutrient competition with tumor cells

Tumor cells outcompete CD8^+^ TILs for glucose in the TME. Low glucose levels enforce TILs to rely on alternative energy sources. From a therapeutic perspective, PD-1 blockade targeting tumor cells could inhibit mTORC1 pathway and further limit glycolysis. Consequently, effector T cells with increased glucose uptake are able to expand their proliferation (3).

A lack of glutamine in the TME could weaken effector T cell activity (47, 158). A prodrug of the glutamine antagonist (6-dizao-5-oxo-L-norleucine) is administrated to a mouse model with colon tumor, leading to decreased glutamine metabolism within the tumor and increased glutamine availability in the TME. Importantly, co-administration of PD1-targeted ICI and glutamine antagonist could induce complete responses and a durable memory-like T cell phenotype (63). Nevertheless, tumors that consume less exogenous glutamine may fail to respond to this combination treatment. Likewise, a low level of extracellular arginine restricts T cell proliferation (159). Compared to ARG1 inhibitor monotherapy, more successes are observed when ARG1 inhibitor is in combination with ICI. Furthermore, IDO1 and catabolite kynurenine are highly expressed in tumor cells to impair T cell fitness by depleting tryptophan and accumulating immunosuppressive metabolites. IDO inhibition, like 1-methyltrytophan, renders the TME less immunosuppressive and facilitates CD8^+^ TIL activity. To increase tryptophan availability, IDO inhibitors combined with ICI are currently being assessed in clinical trials for the treatment of a variety of solid tumors. Besides, kynureninase that can directly catabolize kynurenine, is evidenced to synergize with ICI to extend survival in the preclinical models (160).

Theoretically, memory T cells obtain energy by depending on fatty acids (149). But tumor cells outcompete with memory T cells in fatty acid uptake, thereby inducing apoptosis of memory T cells. This phenomenon could be rescued by PD-1 blockade inhibitor by upregulating fatty acid-binding protein (Fabp) 4/5 in memory T cells and downregulating its expression in tumor cells (161).

## 6 T cell metabolism in the context of ACT

### 6.1 Advantages of ACT to counteract metabolic barriers

In ACT, autologous CD8^+^ TILs targeting tumor-specific antigen or peripheral blood T cells transduced with chimeric antigen receptor T (CAR-T) cells targeting tumor-associated antigen, are expanded *in vitro* and readministered to the patient. ACT is superior to counteract metabolic stress and nutrient deficiency caused by cancer metabolic reprogramming. Therapeutic T cells are activated and expanded *in vitro* under culturing conditions with ample nutrients, overcoming the metabolic challenges that may exist during T cell priming, proliferation, and differentiation, contributing to an augment immunity. ACT provides a unique chance to manipulate T cells before transfer to improve their metabolic fitness. Once transplanted to the recipient, these T cells must infiltrate into the solid tumor and perform effector functions. Therefore, modulating T cell metabolism during ex vivo TIL expansion phase or modifying chimeric antigen receptor design to overcome metabolic barriers would benefit patients with solid tumors (162).

### 6.2 Metabolic modulation in ACT to induce longer-lived memory T cells

CAR-T cell persistence is positively correlated with better tumor clearance and potent anti-tumor response (163), which could be achieved by harvesting longer-lived memory T cells. It has been demonstrated that adoptive T cells derived from central memory T cells can yield a more persistent response *in vivo* compared with those derived from effector cells (164, 165). However, the usual medium for ACT may not support the proliferation of long-lived memory T cells that mainly utilize OXPHOS (166). Explicitly, memory T cells undergo a metabolic shift from glycolysis towards oxidative metabolism motivated by carnitine palmitoyltransferase (CPTA1) (167). Therefore, altering T cell metabolism, by manipulating CAR-T designs or through modifying culture condition, may enforce the memory phenotype to support T-cell persistence and efficacy.

One early study pointed out that mTORC1 inhibition with rapamycin resulted in increased number of memory T cells after pathogen clearance (168). Besides, mTORC2-AKT inhibition during ex vivo expansion can also endow TILs with memory phenotype and enhance anti-tumor response (169, 170). These findings support innovations that integrate metabolic interventions into current ACT immunotherapy.

The culture condition is usually hyper-glycaemia compared with normal human plasma (141). Such a high glucose concentration may induce metabolic stress after infusion. One strategy is to use glycolysis inhibitor 2-DG during the vitro expansion phase, stabilizing a memory-like phenotype after transfer to achieve stronger tumor clearance (45). Modifying ex vivo expansion condition also includes the supply of IL-15 or L-arginine in media to enhance OXPHOS in order to increase the generation of memory T cells (121, 171). Importantly, CAR-T cells cultured with a shorter period may be affected less in hyper-glycaemia medium, giving rise to a subset of less differentiated T cells that have a good proliferative capacity (172).

Genetic manipulation of CAR-T cells with enhanced mitochondrial metabolism can enforce memory phenotype as well. First, 4-1BB-containing CARs support ACT to gain increased mitochondrial biogenesis and FAO, accompanied with better persistence than CD28-containing CARs (173). Second, it is feasible to directly promote metabolism pattern of memory T cells. Upregulating the transcriptional co-activator PGC1*α* or employing the PPAR agonists both mediate enhanced FAO, enriching early memory T cells and improving T cell functionality against tumors (77, 130, 151).

### 6.3 Metabolic interventions in ACT to overcome metabolic barriers imposed by the TME

T cells may be manipulated to acclimatize to low oxygen level in the TME. Currently, CAR-T cell therapy for the treatment of solid tumors is still unsuccessful, partly due to their poor performance under hypoxia. Culturing CAR-T cells in media with a low level of oxygen has been shown to acclimate them to a hypoxic environment and increase their cytotoxic effect on tumor cells (174). It is true T cells would primarily upregulate HIF-1*α* expression in response to the hypoxic signal. In the mice model transplanted with solid tumors, HIF-1*α* deletion resulted in a decreased number of TIL and a heavy tumor burden, verifying the importance of the adaption to hypoxia (175).

Modulation of T cell metabolism can overcome metabolic barriers imposed by TME-specific metabolites as well. Overwhelming evidence suggested metabolite adenosine inhibits the effector function of immune cells (91, 176, 177). Targeting the adenosine A2AR receptor has been confirmed to improve the activity of CD8^+^ T cells (178). Further, the combination of adenosine A2AR receptor antagonist and CAR-T cell therapy is able to increase the efficacy in breast cancer models (179). The release of ions by necrotic tumor cells can disrupt TCR signaling and impair T cell function. For example, high-level of intracellular potassium in T cells can suppress TCR-driven AKT-mTOR signaling (180). Overexpression of the K+ efflux channel in TILs during ex vivo expansion can optimize the treatment efficacy of ACT in melanoma-bearing mice (180).

### 6.4 Metabolic interventions in ACT to overcome nutrient competition with tumor cells

T cells can be engineered to overcome nutrient deficiency in the TME to boost anti-tumor responses. Highly metabolic tumors are more likely to develop resistance to ACT therapy, mainly because nutrient insufficiency can impair T cell trafficking and cytotoxicity (142). Glucose restriction can induce insufficient phosphoenolpyruvate (PEP) and decreased T cell receptor (TCR) signaling in TILs (181). Correspondingly, increasing PEP levels *via* overexpression of phosphoenolpyruvate carboxykinase (PCK1) could improve ACT, supporting intra-tumoral TCR-mediated Ca2^+^-NAFT signaling and T cell effector function (181). As previously introduced, arginine depletion also blocks T cell proliferation. Therefore, engineering CAR-T cells to express arginine synthetase (OTC and ASS) could prompt T cell proliferation and maintenance, along with better efficacy compared with CAR-T cells without these enzymes (182). Additionally, if the culturing condition mimics the TME, these T cells might adapt to low nutrient availability after infusion back to patients. It has been shown that T cells cultured in low-nutrient medium tend to differentiate into a memory phenotype and express less inhibitory receptors (62). For example, T cells in low-glutamine media can produce more potent anti-tumor responses than T cells in traditional media after transfer (62).

## 7 T cell metabolism in the context of oncolytic virus therapy

OV therapy is a form of immunotherapy that uses viruses to selectively identify, infect, and destroy tumor cells, aiming to reduce the tumor progression. Tumor lysis occurs in an immunogenic manner with the production of cytotoxic CD8^+^ T cells infiltrating into the solid tumor (138). So far, only the herpesvirus talimogene laherparepvec (T-VEC) has been approved by the FDA (183). Currently, it is widely accepted that the OV-mediated T cell response is crucial to tumor clearance (184–186). Similar to ACT immunotherapy, OV therapy aims to generate the *de novo* immune response, targeting both tumor and viral antigens released during tumor lysis. However, the *de novo* T cells generated in OV therapy may undergo the same metabolic barriers as observed in T cells activated by ICI or transferred by ACT.

### 7.1 Metabolic modulation to enhance OV therapy

Skewing tumor metabolism towards OXPHOS may enhance OV replication and lytic capacity. Dicholoroacetate (DCA) that inhibits pyruvate dehydrogenase kinase, can shift tumor metabolism to OXPHOS. When tumor-bearing mice were treated with Newcastle disease virus (NDV) in combination with DCA, it is surprising to observe increased NDV replication, along with reduced glucose uptake and lactate production by tumor cells and increased survival in mice compared with NDV therapy alone (187). Oncolytic adenoviruses in combination with the glycolysis inhibitor 2-DG, can get the similar results in preclinical models as well (144). It can be inferred that metabolic modulation may improve cytotoxic CD8^+^ T cell response induced by OV therapy. However, tumors with enhanced oxidative metabolism tend to induce hypoxia in the TME, which can impair CD8^+^ T cell response as priorly discussed. A recent study put forward that, selecting tumors with oxidative metabolism to receive OV therapy, may be superior to metabolic modulation that shift tumor metabolism towards OXPHOS (188). Other metabolic pathways and metabolites are also involved in viral replication and tumor lysis. For example, vaccinia virus needs glutamine and TCA cycle to maintain the viral replication and induce tumor death (145). Moreover, Mevalonate inhibition can render tumor cell more sensitive to oncolytic M1 virus therapy (189). Therefore, metabolic modulators have the potential to increase OV replication and lytic ability, though the mechanisms of action are poorly understood.

### 7.2 Engineering OVs to encode metabolic modulator genes

Oncolytic viruses can be engineered to carry on genes encoding metabolic modulators. For example, engineering vaccinia viruses is able to increase the generation of adipokine leptin by tumor cells after viral infection, leading to the TME remodeling and enhanced anti-tumor response (190). Besides, leptin in the TME can prompt memory precursor T cell differentiation that confers a quick response upon tumor rechallenge (190).

Taken together, the crosstalk between metabolism and OV therapy is a new field that deserves further exploration. Existing findings highlight the importance of identifying the potential OV-responsive patients. And multiple combination immunotherapies are being tested in preclinical models or in clinical trials. The T-VEC is in combination with PD-1 ICI is being explored (191, 192). In solid tumor-bearing preclinical models, OVs are being investigated in combination with ACT therapy (193).

## 8 Conclusion

One can appreciate the fact that the rewiring of cellular metabolism is critical for the initiation and progression of solid tumor. Likewise, such a metabolic transition also occurs in T cells to motivate their priming, activation, differentiation, and functionality. It is widely accepted that T cells utilize glycolysis for cytotoxic function and FAO for immunological memory. However, the TME generally exhibits deprived nutrients, accumulated immunosuppressive metabolites, hypoxia, and acidification, limiting T cell anti-tumor responses. As a result, T cell must undergo metabolism alterations in adaption to the external stimuli. Modulating T cell metabolism pattern in the TME is a promising way of synergy with cancer immunotherapy. As more different immunotherapies into the clinic, interests have focused on T cell metabolism in the context of ICI, ACT, and OV. It remains crucial to understand the basic mechanistic underpinnings to harness immune metabolism. A more comprehensive understanding of immunometabolism would help physicians design combinational strategies or innovations that can overcome metabolic barriers imposed by solid tumor and the TME.

## Author contributions

YQ designed the study and reviewed the manuscript. CC participated in study design and wrote the original draft of the manuscript. CC and ZW was mainly responsible for the design of tables and figures. YD contributed to the conception of the paper. All authors agreed to the submission of the final manuscript.
